# CCDC106 promotes the proliferation and invasion of ovarian cancer cells by suppressing p21 transcription through a p53-independent pathway

**DOI:** 10.1080/21655979.2022.2066759

**Published:** 2022-04-29

**Authors:** Na Zhao, Chen Wang, Peng Guo, Jun Hou, Hong Yang, Ting Lan, Yehan Zhou, Jiayu Li, Ujjal K. Bhawal, Yang Liu

**Affiliations:** a Sichuan Cancer Hospital & Institute, Sichuan Cancer Center, School of Medicine, University of Electronic Science and Technology of China, Chengdu, Sichuan, China; b Department of Histology, Nihon University School of Dentistry at Matsudo, Chiba, Japan; c Department of Pharmacology, Saveetha Dental College, Saveetha Institute of Medical and Technical Sciences, Chennai, India; d Department of Biochemistry and Molecular Biology, Nihon University School of Dentistry at Matsudo, Chiba, Japan

**Keywords:** CCDC106, ovarian cancer, p53, proliferation, invasion

## Abstract

Ovarian cancers are the major cause of mortality for women worldwide. This study was aimed to elucidate the biological activities of CCDC106 in the proliferation and invasion of mutant p53 and of wild-type p53 ovarian cancer cells. CAOV3 (mutant p53) cells showed high expression levels of CCDC106, but it was expressed at low levels in SKOV3 (mutant p53) and in A2780 (wild-type p53) cells. The overexpression of CCDC106 promoted the expression of proliferation markers (cyclin family members), invasion and Epithelial-to-mesenchymal transition (EMT) markers (claudin-1, claudin-4, N-cadherin, snail, slug) while the knockdown of CCDC106 inhibited their expression in mutant p53 cells but not in wild-type p53 cells. Treatment with a CK2 inhibitor blocked the translocation of CCDC106 into the nuclei of mutant p53 cells. Immunoprecipitation assays confirmed that ATF4 is a potential binding partner of CCDC106. The overexpression of CCDC106 reduced p21 and p27 protein expression levels while treatment with an ATF4 siRNA rescued their expression. The overexpression of CCDC106 promoted colony formation and invasion of mutant p53 cells, which was suppressed by treatment with an ATF4 siRNA. Immunohistochemistry results showed that CCDC106 and ATF4 are expressed at high levels but p21 is expressed at low levels in FIGO III–IV stage and in mutant p53 ovarian cancer samples. A significant association between poor overall survival and high CCDC106 and ATF4 expression levels was observed in human ovarian cancer samples. In conclusion, CCDC106 promotes proliferation, invasion and EMT of mutant p53 ovarian cancer cells via the ATF4 mediated inhibition of p21.

## Graphical abstract. Schematic diagram of the biological function of CCDC106 in ovarian cancer. CCDC106 can regulate cyclins and EMT-related markers in the nucleus through ATF4/p21 (p53-independent) pathway. In cytoplasm, CCDC106-induced tumor growth via p53-regulated apoptotic pathway

**Figure:**
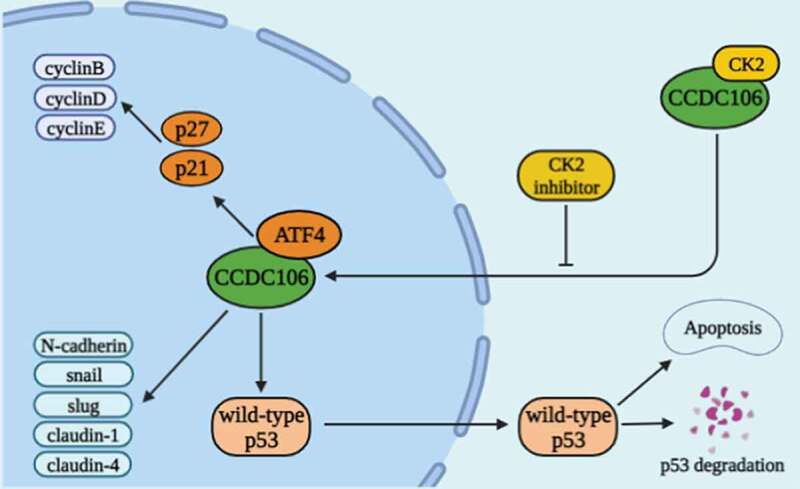

## Highlights

- Nuclear CCDC106 and deficiency of p53 facilitates the progression and invasion of ovarian cancer cells through the ATF4/p21 pathway.
- ATF4 is a potential binding partner of CCDC106.
- Poor overall survival and high CCDC106 and ATF4 expression levels are significantly associated in human ovarian cancers.
- CCDC106 is a potential therapeutic target for patients with p53-deficient ovarian cancers.

## 1. Introduction

Coiled-coil domain containing 106 (CCDC106) is a protein containing 280 amino acids with a molecular weight of 32 kDa [1]. The coiled-coil domain is involved in molecular recognition, DNA binding and secretion. CCDC106 was originally discovered in a yeast two-hybrid screen, but little is known about its biological function(s) and mechanism(s).

It has been reported that the functional status of p53 is closely related to tumor prognosis and progression [2–4]. As a cell cycle regulatory gene and an important tumor suppressor gene, p53 plays a key role in the DNA damage response and in tumor signaling pathways [5–7]. p53 is dysregulated in most cancers [8,9], usually due to mutations in the p53 gene, epigenetic changes and/or p53 metabolic abnormalities [10,11]. In wild-type p53 tumors, p53 is usually inactive [12], which may be virus-induced, e.g., by the human papillomavirus E6 oncoprotein [13], due to the up-regulation of its transcriptional repressors, murine double minute (MDM) 2 and MDMX [14–19], or due to accelerated degradation of the p53 protein [20]. For wild-type p53 tumors in which p53 is inactive, cells lack the G1 phase checkpoint, which monitors DNA damage and maintains the integrity of the genome. Thus, those cells avoid p53-induced apoptosis, retain mutations and promote tumorigenesis [21,22]. In a prior study, Zhou et al. showed that CCDC106 exerts its effects by promoting the degradation of p53 [20]. They speculated that CCDC106 plays a role in promoting the growth of wild-type p53 tumors but does not affect the evolution of mutant p53 tumors. Our previous study demonstrated that CCDC106 promotes AKT phosphorylation, which promotes cyclin upregulation and cell proliferation in the mutant p53 lung cancer cell line, H1299 [23]. Therefore, we hypothesized that for tumors with p53 deletions, there must be a p53-independent pathway that regulates tumor functions via CCDC106.

p21 has been reported to suppress ovarian cancers through the induction of apoptosis [24] and positive p21 expression resulted in an increased link to overall survival in patients with ovarian cancers [25]. As a negative regulator of the cell cycle, p27 is downregulated in many types of cancers [26]. In addition, Bali et al. reported that the increased expression of cyclin D1 and p53, and the reduced expression of p21 and p27 are connected to a poor prognosis for patients with ovarian cancers [27,28]. The overexpression of cyclin E1 is associated with tumorigenesis and reduced overall survival of ovarian cancer patients [29]. The epithelial-mesenchymal transition (EMT) characterizes multiple biochemical alterations in epithelial cells and the motility and invasive behaviors of the mesenchymal phenotype [30]. The dynamic features of ovarian cancers in the context of the EMT are a fundamental challenge [31]. A previous study demonstrated that E-cadherin is highly expressed in well-differentiated ovarian tumors, while N-cadherin is upregulated in advanced stage and metastatic tumors [32]. CCDC106 has two phosphorylation sites, S130 and S147, which bind to the CK2β subunit of Casein kinase II (CK2 kinase) and are then phosphorylated by CK2 kinase [33]. This phosphorylation is essential for CCDC106 to enter the nucleus. CCDC106 interacts with p53 in the nucleus and facilitates the degradation of p53. Decreased p53 nuclear levels regulate the cell cycle and apoptosis through the B-cell lymphoma (BCL)-2 pathway [33]. Based on these data, we hypothesized that the subcellular localization of CCDC106 affects its biological function(s).

To study the correlation between the subcellular localization and biological influences of CCDC106 in ovarian cancer cells, and to characterize the mechanism through which CCDC106 facilitates the progression of ovarian cancer cells, we selected cell lines in which CCDC106 was localized either to the nucleus or to the cytoplasm. We used CK2 inhibitors to block the entry of CCDC106 into the nucleus to validate our findings and utilized a mutant p53 cell line to identify possible regulatory pathways that are controlled by nuclear CCDC106. Our results enhance understanding of the molecular mechanism of CCDC106 in cancer cells, particularly its role in mutant p53 tumors, and identify CCDC106 as a potential therapeutic target in patients with mutant p53 cancers.

## 2. Materials and methods

### 2.1 Patients and clinical records

This study was conducted in accordance with the approval of the Institutional Review Board of the Sichuan Cancer Hospital (Approval Number SCCHEC-02-2022-011). A total of 119 surgically excised ovarian cancer specimens with a final diagnosis of High grade serous, Clear cells or Endometrioid were collected from patients at the Sichuan Cancer Hospital, with complete follow-up data. Neoadjuvant chemoradiotherapy cases were excluded from this study.

### 2.2 Cell culture

Mutant p53 (SKOV3, OVCAR3) and wild-type p53 (A2780) ovarian cancer cell lines were obtained from the ATCC (Manassas, VA, USA) and were cultured in RPMI-1640 (Invitrogen, Carlsbad, CA, USA) supplemented with 10% fetal bovine serum FBS (Invitrogen, Carlsbad, CA, USA), 100 IU/ml penicillin (Sigma, St. Louis, MO, USA) and 100 μg/ml streptomycin (Sigma, St. Louis, MO, USA) in 5% CO_2_ at 37°C. The mutant p53 (CAOV3) ovarian cancer cell line was obtained from the Shanghai Cell Bank (Shanghai, China) and was cultured in DMEM (Invitrogen, Carlsbad, CA, USA).

### 2.3 Western blotting

Lysis buffer (Pierce, Rockford, IL, USA) was used to extract total protein. Fifty μg of each protein sample were separated on SDS-PAGE, and were transferred onto polyvinylidene fluoride membranes (PVDF, Millipore, Billerica, MA, USA). Primary antibodies used targeted: CCDC106 (1:1000, ab105354, Abcam, Cambridge, UK), GAPDH (1:1000, Sigma, St. Louis, MO, USA), ATF4 (1:1000, Proteintech, Wuhan, China) and Cyclin A2, Cyclin B1, Cyclin D1, Cyclin D2, Cyclin D3, Cyclin E1, Cyclin E2, Cyclin H, p21, p27, E-cadherin, N-cadherin, Vimentin, Slug, Snail, Claudin-1, Claudin-4, MMP2, MMP7, MMP9 and ZEB1 (1:1000; all from Cell Signaling Technology, Danvers, MA, USA). Antibodies were incubated with the PVDF membranes overnight at 4°C. After washing the membranes with PBS, appropriate secondary antibodies, peroxidase-conjugated anti-mouse or anti-rabbit IgG (1:5000, Santa Cruz Biotechnology, Inc., Santa Cruz, CA, USA) were incubated at 37°C for 2 h. Electrochemiluminescence (Pierce, Rockford, IL, USA) and a bio-imaging system (DNR BioImaging Systems, Jerusalem, Israel) were used to detect and analyze the bound antibodies.

### 2.4 Immunohistochemistry

The formalin-fixed and paraffin-embedded tissues were cut into sections at a thickness of 4 μm. Antibodies were incubated overnight at 4°C at the following dilutions: 1:200 for CCDC106 (Abcam, Cambridge, MA, USA); 1:100 for p53 (Cell Signaling Technology, Danvers, MA, USA); 1:200 for p21 (Proteintech, Wuhan, China) and 1:500 for ATF4 (Proteintech, Wuhan, China). A biotin-labeled secondary antibody (Ultrasensitive; MaiXin, Fuzhou, China) was incubated at room temperature for 30 min, and color was developed using 3,3’-diaminobenzidine (DAB) Chromogen solution (Dako, Tokyo, Japan).

The expression of CCDC106, p53, p21 and ATF4 was scored according to the number of positively stained cells and their staining intensity. Expression was divided into four grades according to the color intensity: 0 (no color), 1 (light yellow particles), 2 (medium strength yellow particles) and 3 (dark yellow or tawny particles). According to the staining area of cells, there were four grades: 1 (1%-25%), 2 (26%-50%), 3 (51%-75%) and 4 (76%-100%). A final score of 0–12 was obtained by multiplying the intensity and percentage scores.

### 2.5 Immunofluorescence staining

Cells were cultured in chamber slides overnight, then fixed with ice-cold methanol at −20°C for 10 min followed by permeabilization with 0.2% Triton X-100. Cells were then incubated with normal sheep serum for 30 min (to minimize nonspecific binding of antibodies) followed by incubation with anti-CCDC106 (1:50, Abcam, Cambridge, UK) or anti-ATF4 (1:50, Proteintech, Wuhan, China) antibodies at 4°C overnight. Goat anti-rabbit or anti-mouse IgG antibodies (1:1000, Proteintech, Wuhan, China) were used as secondary antibodies at room temperature for 2 h. Cells were stained with DAPI (Thermo Fisher Scientific, Tokyo, Japan) and a fluorescence microscope was used to acquire images.

### 2.6 Plasmid transfection and small interfering RNAs

The empty plasmid, CCDC106 plasmid, ATF4 siRNA (Origene, Rockville, MD, USA) and CCDC106 siRNA, Scramble siRNA, p53-shRNA, Scramble shRNA (Santa Cruz Biotechnology Inc., Santa Cruz, CA, USA) were used in this study. Transfection using Lipofectamine 3000 reagent (Invitrogen, Carlsbad, CA, USA) was carried out according to the manufacturer’s protocol.

### 2.7 CCK8 assay

Cells were harvested 24 h after transfection and seeded in 96-well plates (3,000 cells per well). Three replicate wells were set for each group, and cells were collected at 24 h, 48 h, 72 h, 96 h and 120 h for absorbance detection. Results were plotted with the horizontal axis showing time and the vertical axis indicating absorbance to draw a standard curve and for the analysis of data. The CCK8 assay was also employed to determine the optimal concentration of the CK2 kinase inhibitor (MedChemEXpress, LLC, Monmouth Junction, NJ, USA) in SKOV3 cells for 24 h, followed by subsequent analyses.

### 2.8 Colony formation assay

Cells were harvested 24 h after transfection and seeded in 6-cm dishes (1,000 cells per dish) for 12 days. The culture medium was replaced every 4 days. The cells were fixed with 4% paraformaldehyde for 10 min followed by incubation in hematoxylin. The colony numbers of more than 50 cells were counted.

### 2.9 Transwell migration assays

Transwell chambers containing 8 µm pores were placed in 24-well plates. One × 10^5^ cells supplemented with 2% serum in 100 µl medium were seeded into the upper chamber 24 h after transfection. The lower chamber contained 20% serum in 600 µl medium. After 20 h of incubation, the cells were fixed with 4% paraformaldehyde for 10 min followed by incubation in hematoxylin. The upper membrane surface was wiped off with cotton swabs to remove noninvasive cells. Ten randomly selected field views were used to count the number of invading cells.

### 2.10 RNA extraction and Real-time RT-PCR

RNAiso plus (Qiagen, Hilden, Germany) was used to lyse the cells to be tested. RNA extraction was performed using chloroform and isopropanol followed by washing with ethanol. Residual ethanol was carefully absorbed with filter paper and the precipitates were dried in air. After that, an appropriate amount of RNAse-free water was added to dissolve the precipitate. A PrimeScript^TM^ RT reagent Kit with gDNA Eraser (TOYOBO, Osaka, Japan) Kit was used to synthesize complimentary DNA and SYBR Green PCR master matrix (Applied Biosystems, Tokyo, Japan) was used for quantitative real-time PCR. Relative gene expression levels were expressed using the ∆Ct = Ct gene – Ct reference and were calculated using the 2-∆Ct method. The experiments were conducted in triplicate, and GAPDH was used as a housekeeping gene. The primer sequences of real-time quantitative PCR were as follows: ATF4: forward (GTCCTCCACTCCAGATCATTCC); reverse (AGGACTCT GGGCTCATACAGAT); p21: forward (GGGACAGCAGAGGAAGAC); reverse (TGGAGTGGTAGAAATCTGTCA); GAPDH: forward (TGAAGGTCGGAGTCAACGGATTTGGT); reverse (CATGTGGGCCATGAGGTCCACCAC) [34–36].

### 2.11 Isolation of nuclear and cytoplasmic proteins

SKOV3 and A2780 cells were harvested 48 h after transfection with CCDC106 and a NE-PER Nuclear Cytoplasmic Extraction Reagent kit (Thermo Fisher Scientific, Waltham, MA, USA) was used to isolate nuclear and cytoplasmic proteins, in accordance with manufacturer’s instructions.

### 2.12 Immunoprecipitation analysis

The cell lysates were centrifuged at 4°C at 12,000 rpm for 15 min. The supernatants were concentrated, and their protein concentrations were calculated. The cell lysates were placed on ice and incubated with magnetic beads overnight. Antibodies were then incubated using a rotating shaker at 4°C overnight and the immune complexes were collected for analysis by western blotting.

### 2.13 Statistical analysis

All statistical analyses were performed using GraphPad Prism 7.0 and SPSS 23.0. One-way ANOVA, the Mann-Whitney U test and a ratio paired t-test were used to compare differences among the groups. A p value of less than 0.05 was considered statistically significant.

## 3. Results

### 3.1 Expression and subcellular localization of CCDC106 in ovarian cancer cells

**Figure 1.**
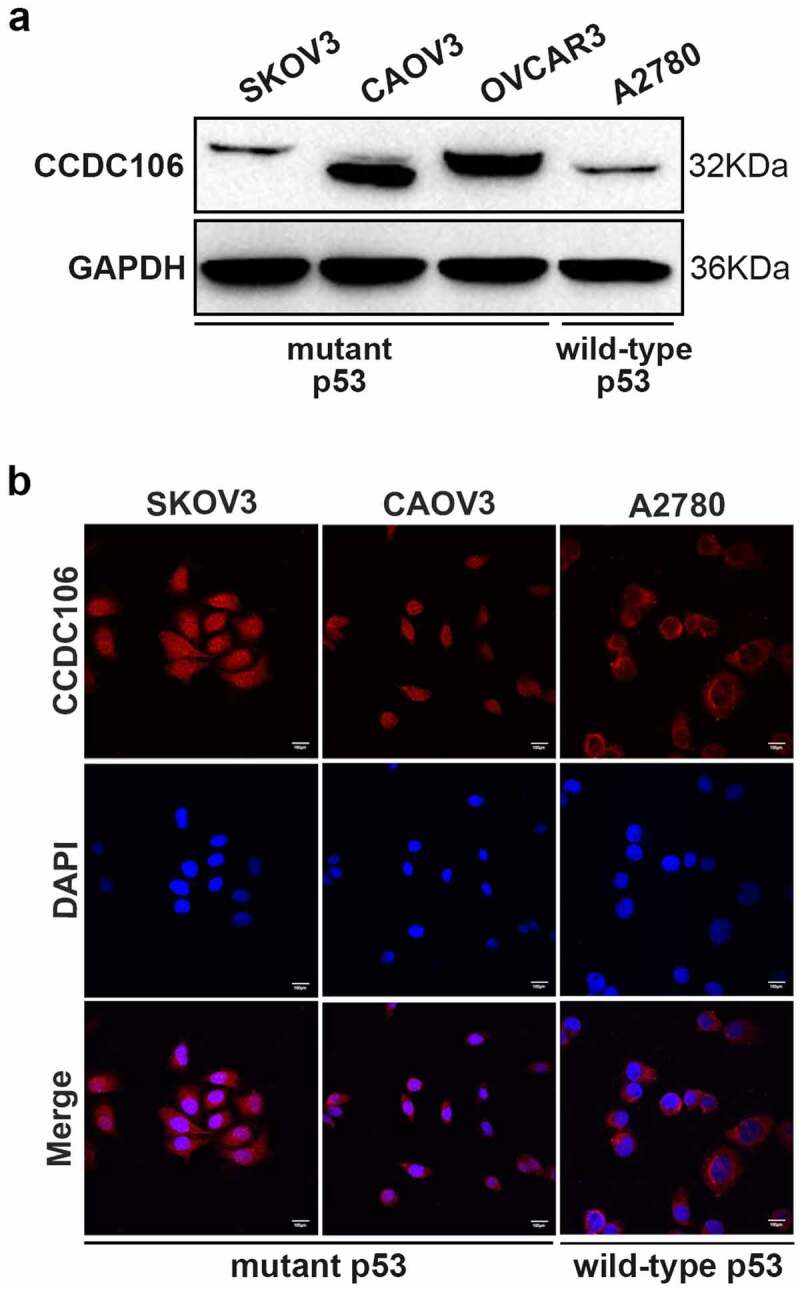
Protein expression levels and subcellular distribution of CCDC106 in ovarian cancer cells. (a, b) Expression levels and localization of CCDC106 in mutant p53 (SKOV3, CAOV3, OVCAR3) and in wild-type p53 (A2780) ovarian cancer cell lines assessed by Western blot and immunohistochemistry, respectively. Scale bars = 100 μm. All experiments were repeated three times with similar results.

First, we confirmed the protein expression level and subcellular distribution of CCDC106 in three mutant p53 (SKOV3, CAOV3, OVCAR3) and in one wild-type p53 (A2780) ovarian cancer cell lines using western blot and immunofluorescence analysis. CCDC106 was expressed at high levels in CAOV3 and OVCAR3 cells but at low levels in SKOV3 and A2780 cells (Figure 1a). Immunofluorescence analysis showed that CCDC106 was predominantly localized in the nuclei of SKOV3 and CAOV3 cells but in the cytoplasm of A2780 cells (Figure 1b). Considering these results, we selected SKOV3 cells for overexpression experiments and CAOV3 cells for knockdown experiments.

### 3.2 Effect of CCDC106 on the proliferation of mutant or wild-type p53 cells

**Figure 2.**
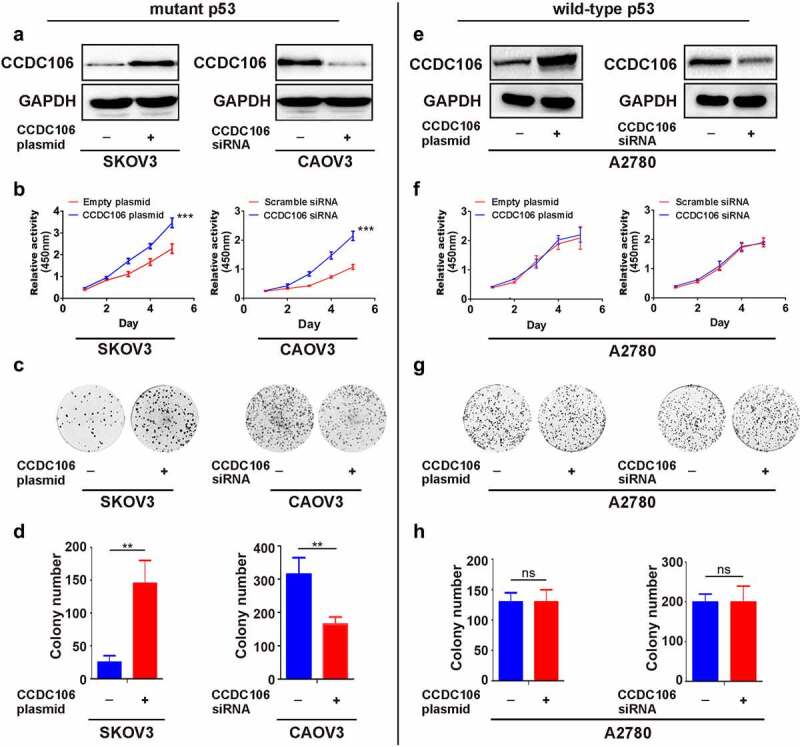
**Effects of CCDC106 on the proliferation of mutant or wild-type p53 ovarian cancer cells**. (a) Representative western blot analyses of CCDC106 overexpression or knockdown in SKOV3 and CAOV3 cells, respectively. (b) Effects of the overexpression or knockdown of CCDC106 on the proliferation of SKOV3 and CAOV3 cells, respectively, assessed by CCK8 assays. (c, d) Effects of the overexpression or knockdown of CCDC106 on colony formation of SKOV3 and CAOV3 cells, respectively. (e) Representative western blot analysis of CCDC106 overexpression or knockdown in A2780 cells. (f) Effects of the overexpression or knockdown of CCDC106 on the proliferation of A2780 cells assessed by CCK8 assays. (g, h) Effects of the overexpression or knockdown of CCDC106 on colony formation of A2780 cells. All experiments were repeated three times. Data represent means ± SD of three independent experiments: **p < 0.01, ***p < 0.001.

CCDC106 overexpression and knockdown experiments were employed to examine the role of CCDC106 in the proliferation of ovarian cancer cells. Transfection efficiencies were evaluated in mutant p53 and in wild-type p53 cells by western blot analysis (Figure 2a, e). CCK8 assays verified that the overexpression of CCDC106 enhanced the proliferation of SKOV3 (mutant p53) cells, while silencing CCDC106 inhibited the proliferation of CAOV3 (mutant p53) cells (Figure 2b). Similar findings were indicated in colony formation assays (Figure 2c, d). However, enhancing or inhibiting CCDC106 expression had no effect on proliferation (figure 2f) or colony formation (Figure 2g, h) in A2780 (wild-type p53) ovarian cancer cells.

**Figure 3.**
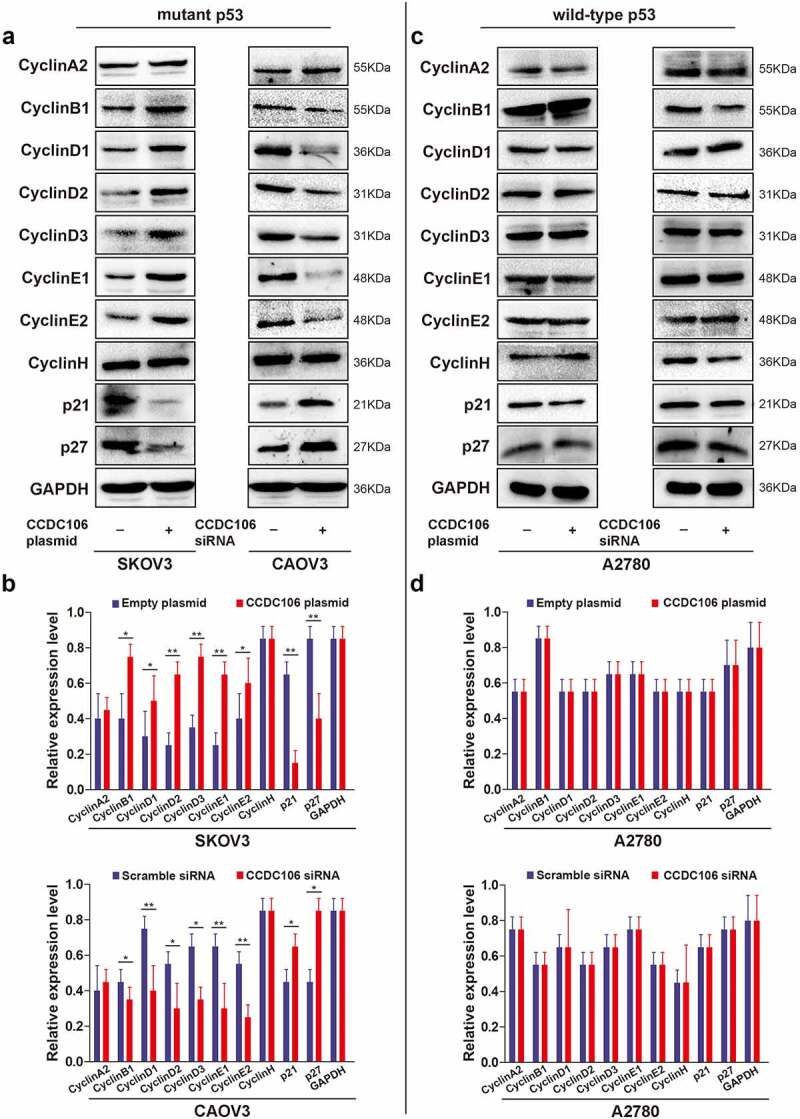
Effects of CCDC106 on the expression of cyclin-related markers in mutant and in wild-type p53 ovarian cancer cells. (a, b) Representative western blot analysis of the effects of overexpression or knockdown of CCDC106 cyclin-related protein levels in SKOV3 and CAOV3 cells, respectively. (c, d) Representative western blot analysis of the effects of overexpression or knockdown of CCDC106 cyclin-related protein levels in A2780 cells. All experiments were repeated three times. Data represent means ± SD of three independent experiments: *p< 0.05, **p < 0.01.

Next, we examined the cellular levels of cyclin-related proteins using western blot analysis. In SKOV3 (mutant p53) cells, the overexpression of CCDC106 upregulated the expression of cyclin B1, cyclin D1, cyclin D2, cyclin D3, cyclin E1 and cyclin E2 while the expression of p21 and p27 was downregulated (Figure 3a, b). Opposite results were observed when CCDC106 was silenced in CAOV3 (mutant p53) cells (Figure 3a, b). However, in A2780 (wild-type p53) cells, CCDC106 had no apparent effect on the expression levels of cyclin-related proteins (Figure 3c, d). These results suggested that the overexpression of CCDC106 promoted the proliferation of mutant p53 ovarian cancer cells, while silencing CCDC106 inhibited it. On the contrary, CCDC106 had no effect on the proliferation of wild-type p53 ovarian cancer cells.

### 3.3 Effect of CCDC106 on invasion and EMT in mutant or wild-type p53 cells

**Figure 4.**
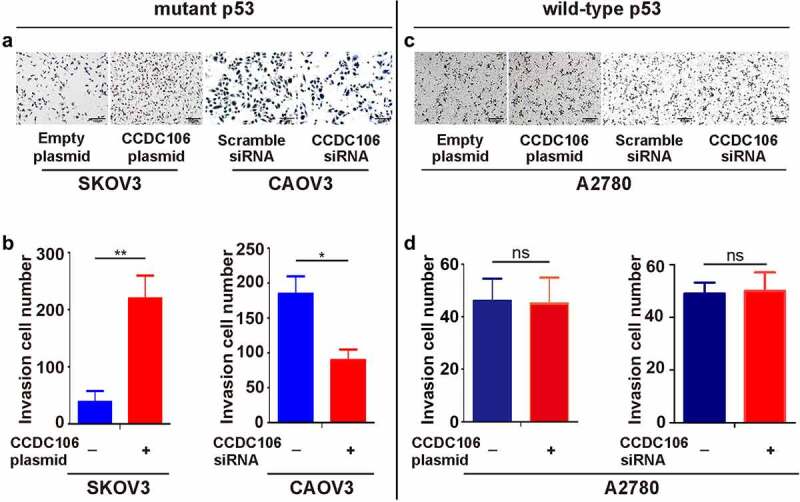
Effect of CCDC106 on the invasion of mutant and of wild-type p53 ovarian cancer cells. (a, b) Effects of the overexpression or knockdown of CCDC106 on the invasion of SKOV3 and CAOV3 cells, respectively, assessed by Transwell migration assays. (c, d) Effects of the overexpression or knockdown of CCDC106 on the invasion of A2780 cells, assessed by Transwell migration assays. Scale bars = 100 μm. All experiments were repeated three times. Data represent means ± SD of three independent experiments: *p < 0.05, **p< 0.01.

To evaluate the importance of CCDC106 in the invasion and EMT of ovarian cancers, Transwell migration assays and western blot analyses were performed after transfection with a CCDC106 plasmid or siRNA in mutant or wild-type p53 cells. The overexpression of CCDC106 in SKOV3 (mutant p53) cells promoted invasion (Figure 4a, b) and the knockdown of CCDC106 had the opposite effect on CAOV3 cells (Figure 4a, b). However, in wild-type p53 A2780 cells, CCDC106 had no effect on invasion (Figure 4c, d).

**Figure 5.**
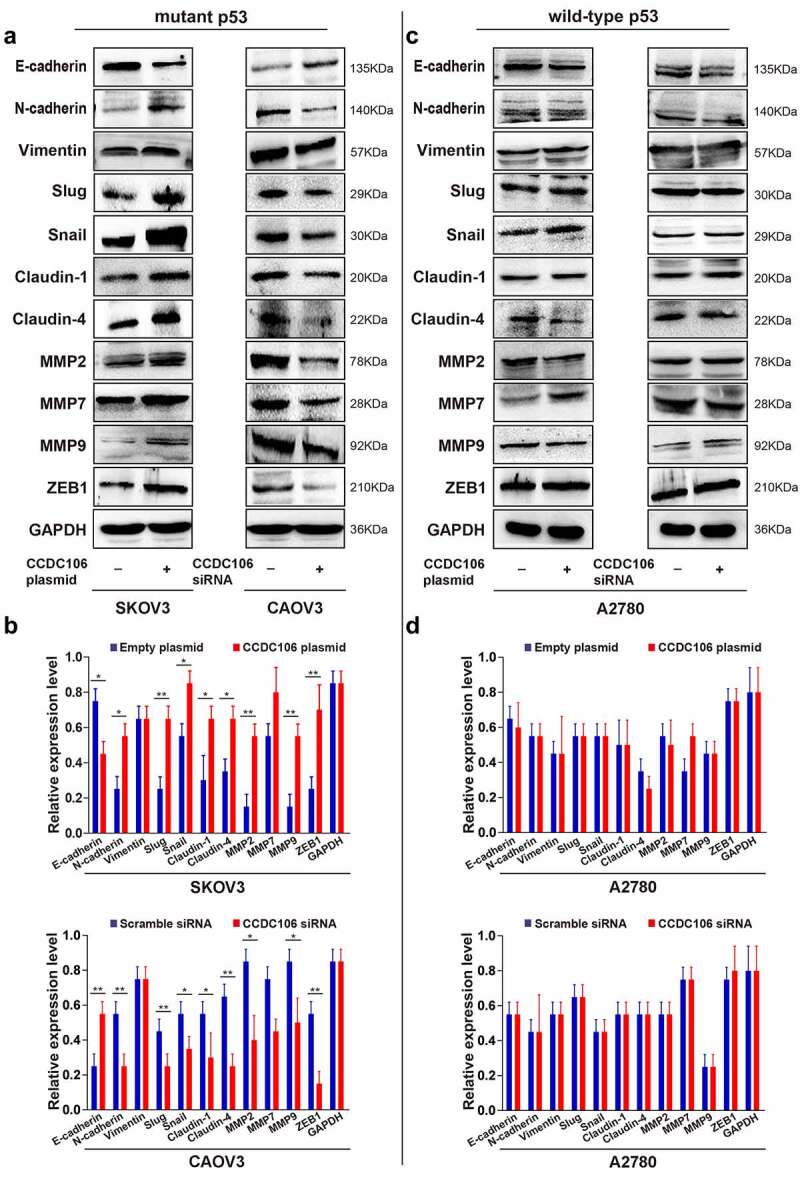
Effects of CCDC106 on the expression of EMT-related markers in mutant and in wild-type p53 ovarian cancer cells. (a, b) Representative western blot analysis of the effects of overexpression or knockdown of CCDC106 EMT-related protein levels in SKOV3 and CAOV3 cells, respectively. (c, d) Representative western blot analysis of the effects of overexpression or knockdown of CCDC106 EMT-related protein levels in A2780 cells. All experiments were repeated three times. Data represent means ± SD of three independent experiments: *p< 0.05, **p < 0.01.

We also examined the protein expression levels of EMT-related markers. We found that overexpression of CCDC106 upregulated the protein expression levels of N-cadherin, slug, snail, claudin-1 and claudin-4 but suppressed E-cadherin expression in mutant p53 SKOV3 cells (Figure 5a, b). Opposite results were obtained when CCDC106 was knocked down in CAOV3 cells (Figure 5a, b). Enhancing or inhibiting CCDC106 expression had no influence on the protein expression levels of EMT-related markers in A2780 (wild-type p53) cells (Figure 5c, d). Thus, our data suggested that the presence of p53 may impact the biological activity of CCDC106.

### 3.4 Translocation of CCDC106 in mutant p53 and wild-type p53 cells

**Figure 6.**
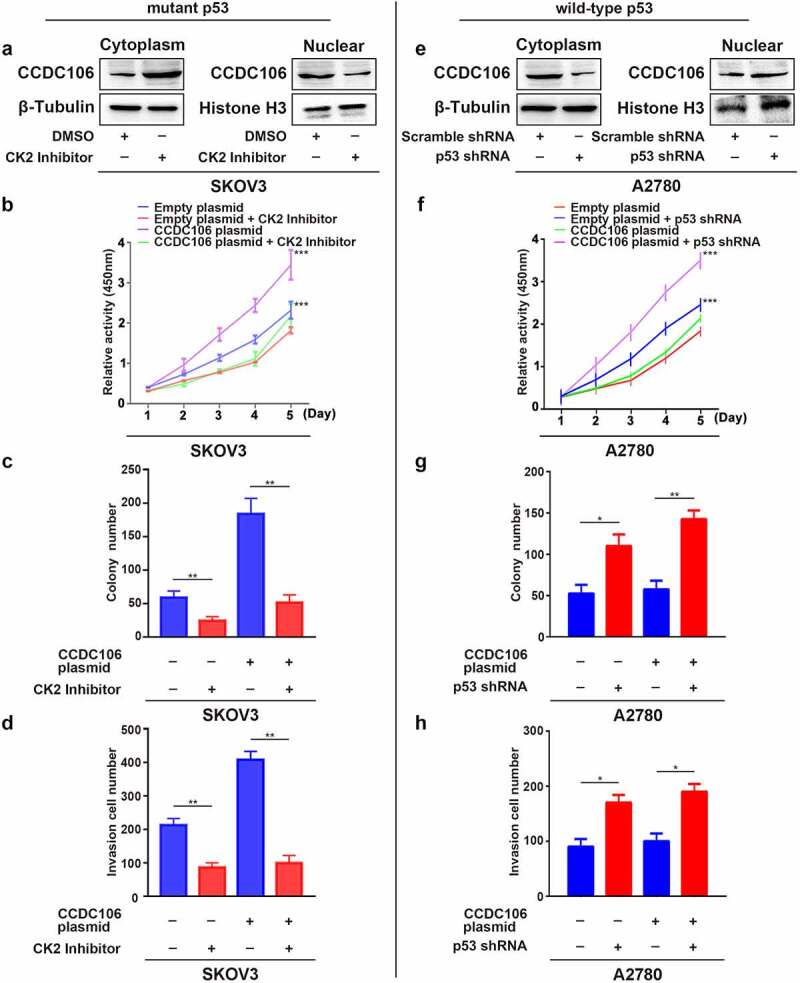
Translocation of CCDC106 to nuclear in mutant p53 and in wild-type p53 ovarian cancer cells. (a, e) Effects of a CK2 kinase inhibitor and p53 shRNA on the subcellular localization of CCDC106 in SKOV3 and A2780 cells, respectively, assessed by nuclear/cytoplasmic fractionation. (b, f) Effects of a CK2 kinase inhibitor and p53 shRNA on the proliferation of SKOV3 and A2780 cells, respectively, assessed by the CCK8 assay. (c, g) Effects of a CK2 kinase inhibitor and p53 shRNA on the proliferation of SKOV3 and A2780 cells, respectively, assessed by colony formation assays. (d, h) Effects of a CK2 kinase inhibitor and p53 shRNA on the invasion of SKOV3 and A2780 cells, respectively, assessed by Transwell migration assays. All experiments were repeated three times. Data represent means ± SD of three independent experiments: *p < 0.05, **p < 0.01, ***p < 0.001.

Next, we examined the nuclear translocation of CCDC106 using a CK2 inhibitor in SKOV3 (mutant p53) cells and employed a p53 shRNA in A2780 (wild-type p53) cells. Western blot analysis confirmed that the CK2 inhibitor effectively blocked the translocation of CCDC106 into the nuclei of SKOV3 (mutant p53) cells (Figure 6a). As expected, treatment with the CK2 inhibitor significantly inhibited the proliferation of SKOV3 cells and inhibited the proliferation induced by the overexpression of CCDC106 (Figure 6b), Colony formation assays (Figure 6c), and Transwell migration assays (Figure 6d).

We further evaluated whether the presence of p53 influences the biological activity of CCDC106. To verify our hypothesis, we assessed the localization of CCDC106 as well as its effect on proliferation, colony formation and invasion in A2780 wild-type p53 cells after the knockdown of p53. Western blot results showed that the silencing of p53 decreased CCDC106 protein levels in the cytoplasm and enhanced them in the nuclei (Figure 6e). CCDC106, which is primarily expressed in the cytoplasm, was localized in the nuclei after knockdown of p53 in A2780 (wild-type p53) cells (Supplementary Figure 1). A p53 deficiency resulted in the increased proliferation of A2780 cells and the overexpression of CCDC106 enhanced that effect (figure 6f). Similar results were obtained in colony formation assays (Figure 6g), and Transwell migration assays (Figure 6h).

### 3.5 Nuclear CCDC106 and deficiency of p53 facilitates the progression of ovarian cancer cells through the Activating Transcription Factor (ATF) 4/p21 pathway

**Figure 7.**
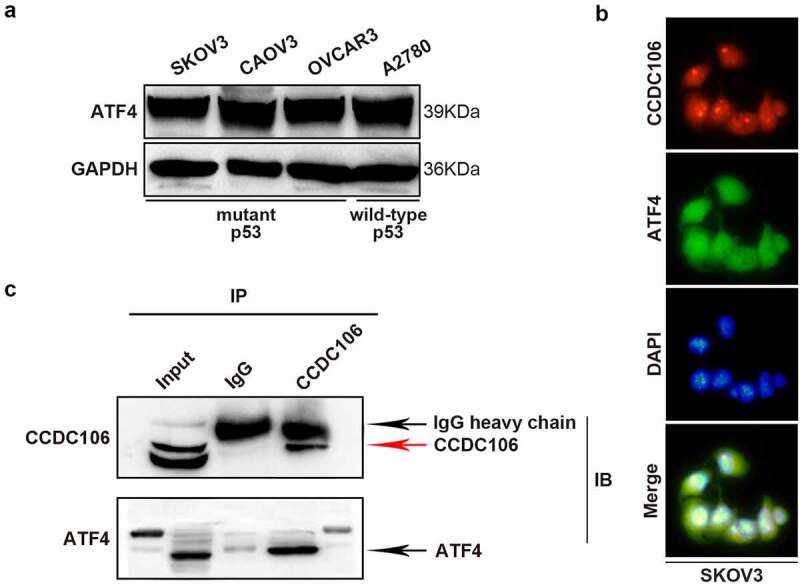
Functional regulation of CCDC106 and ATF4 in mutant p53 ovarian cancer cells. (a) Representative western blot analysis of ATF4 expression levels in ovarian cancer cells. (b) Colocalization of CCDC106 and ATF4 in SKOV3 cells by immunofluorescence. (c) CCDC106 binds to ATF4 in SKOV3 cells in immunoprecipitation results. All experiments were repeated three times with similar results.

**Figure 8.**
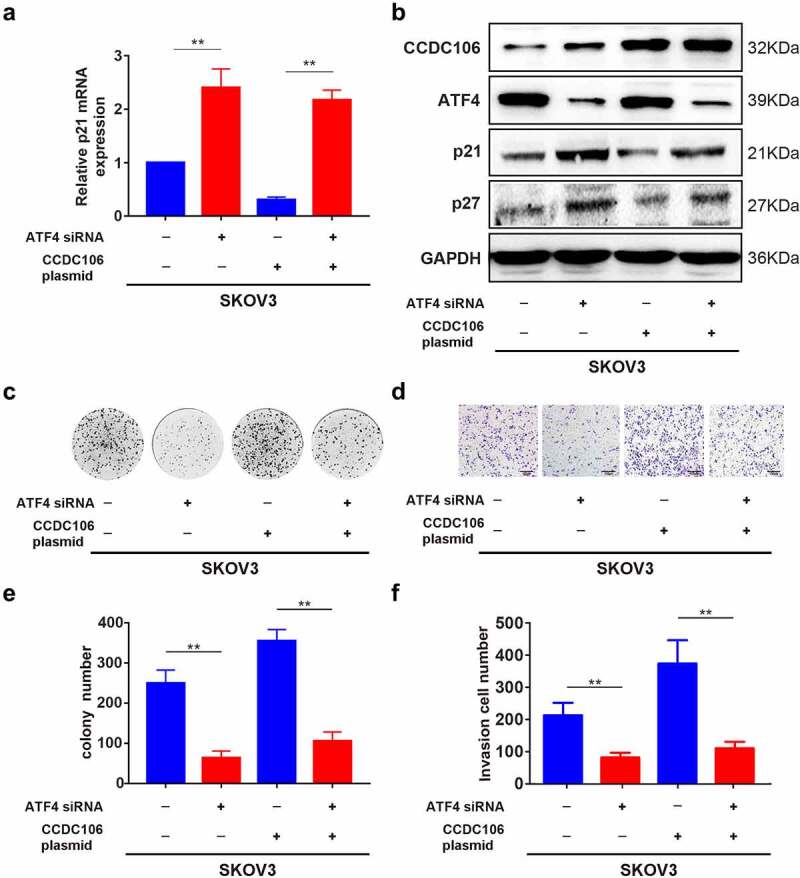
Effects of ATF4 and CCDC106 on p21 and p27 expression in mutant p53 ovarian cancer cells. (a)The effects of ATF4 siRNA or CCDC106 plasmid in SKOV3 cells on levels of p21 mRNA assessed by RT-PCR. (b) p21/p27 protein expression levels assessed by western blot analysis after transfection of SKOV3 cells with an ATF4 siRNA or a CCDC106 plasmid. (c-f) The effects of ATF4 siRNA or CCDC106 plasmid on colony formation and invasion in SKOV3 cells. Scale bars = 100 μm. All experiments were repeated three times. Data represent means ± SD of three independent experiments: **p< 0.01.

ATF4 was selected as a target of CCDC106 by mass spectrometry and alignment analysis (Supplementary Figure 2). We confirmed the expression level of ATF4 in all four ovarian cancer cell lines using Western blot analysis (Figure 7a). SKOV3 (mutant p53) cells were used for subsequent analysis. Immunofluorescence results demonstrated the colocalization of ATF4 and CCDC106 in SKOV3 cells (Figure 7b). Immunoprecipitation assays further verified that ATF4 is a potential binding partner of CCDC106 (Figure 7c). The overexpression of CCDC106 reduced p21 mRNA levels, while treatment with an ATF4 siRNA rescued the expression of p21 (Figure 8a). The expression of p21 and p27 proteins also showed similar results in western blot analysis (Figure 8b). Similar results were obtained in colony formation assays (Figure 8c, e) and in Transwell migration assays (Figure 8d, f).

### 3.6 Immunohistochemical analysis of CCDC106, p53, p21 and ATF4 expression in ovarian cancer tissues

**Figure 9.**
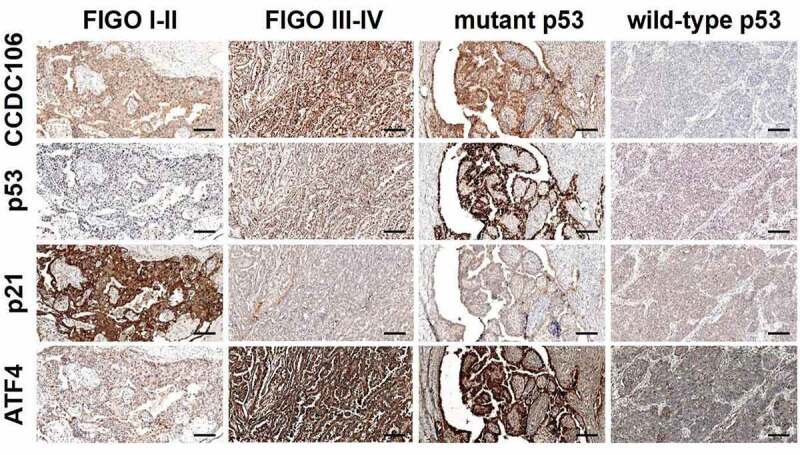
Immunohistochemical analysis of CCDC106, p53, p21 and ATF4 expression levels in ovarian cancer tissues. CCDC106, p53 and ATF4 were weakly expressed in FIGO I-II stage ovarian cancer samples while p21 was positively expressed. In FIGO III-IV stage and mutant p53 ovarian cancer samples, CCDC106, p53 and ATF4 were positively expressed in the nucleus and cytoplasm, respectively, while p21 was weakly expressed in FIGO III-IV stage and in mutant p53 ovarian cancer samples. Scale bars = 50 μm.

We then performed immunohistochemical staining to evaluate the expression of CCDC106, p53, p21 and ATF4 in 119 ovarian cancer tissue samples. Immunohistochemical staining showed that CCDC106, p53 and ATF4 were weakly expressed in FIGO I–II stage ovarian cancer samples while p21 was strongly expressed (Figure 9). In FIGO III–IV stage and in mutant p53 ovarian cancer samples, CCDC106, p53 and ATF4 were positively expressed in the nucleus and cytoplasm, respectively, while p21 was weakly expressed in FIGO III–IV stage and in mutant p53 ovarian cancer samples (Figure 9).

### 3.7 Protein expression levels of CCDC106, p53, p21 and ATF4 correlate with overall survival and clinicopathological parameters in patients with ovarian cancers

**Figure 10.**
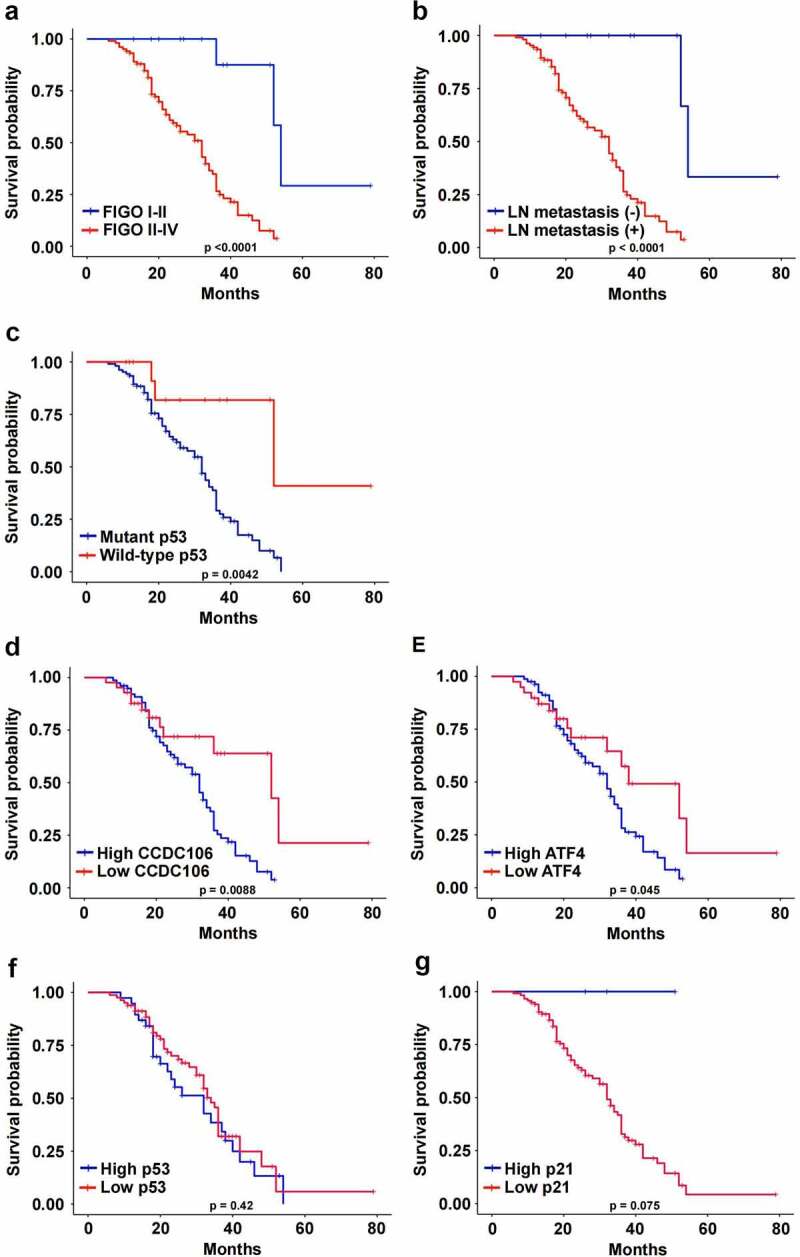
Correlations between CCDC106, p53, ATF4 and p21 expression and overall survival of ovarian cancer patients. (a-c) The overall survival of ovarian cancer patients was significantly correlated with FIGO stage (p<0.0001), lymph node metastasis (p<0.0001) and p53 status (p=0.0042). (d, e) The overall survival of ovarian cancer patients with a high level of CCDC106 and ATF4 was significantly lower than patients with a low level of CCDC106 (p=0.0088) and ATF4 (p=0.045), respectively. (f, g) The overall survival of ovarian cancer patients with high or low expression levels of p53 and p21 was not significantly different.

The correlations between the protein expression levels of CCDC106, p53, p21 and ATF4 and the clinicopathological parameters in human ovarian cancer samples are outlined in Table 1. CCDC106 and ATF4 protein expression were significantly correlated with FIGO stage (p < 0.0001; p = 0.0007), lymph node metastasis (p = 0.009; p = 0.003) and p53 status (p = 0.015; p = 0.008), respectively. FIGO stage (p = 0.0003), lymph node metastasis (p = 0.033) and p53 status (p = 0.015) were also correlated with the expression of p21. p53 expression showed no significant association with any clinicopathological parameters. We also analyzed associations between the survival time of ovarian cancer patients and clinicopathological parameters and the expression levels of CCDC106, p53, p21 and ATF4. The overall survival of ovarian cancer patients was closely associated with FIGO stage (p < 0.0001), lymph node metastasis (p < 0.0001) and p53 status (p = 0.0042) (Figure 10a-c). In addition, the overall survival of ovarian cancer patients with high levels of CCDC106 and ATF4 was significantly lower than patients with low levels of CCDC106 (p = 0.0088) and ATF4 (p = 0.0045) (Figure 10d, e). The overall survival of ovarian cancer patients expressing p53 and p21 was not significantly different (Figure 10f, g).

**Table 1.** Correlation of CCDC106, p53, ATF4 and p21 expression with clinicopathological features in ovarian cancer cases

Clinicopathological factors	N	CCDC106 expression, n (%)				P-value	p53 expression, n (%)				P-value
		Score≤6		Score>6			Score≤6		Score>6		
Total	119	42	(35.3)	77	(64.7)		78	(65.5)	41	(34.5)	
Age (years)						0.0515					0.0608
<50	39	9	(7.6)	30	(25.2)		21	(17.6)	18	(15.1)	
≥50	80	33	(27.7)	47	(39.5)		57	(47.9)	23	(19.3)	
FIGO stage						0.0001					0.3924
I–II	16	13	(10.9)	3	(2.5)		12	(10.1)	4	(3.4)	
III–IV	103	29	(24.4)	74	(62.2)		66	(55.5)	37	(31.2)	
Tumor histology						0.2201					0.1683
High grade serous	112	38	(32.0)	74	(62.2)		72	(61.0)	40	(33.6)	
Clear cells	3	0	(0)	3	(2.5)		3	(2.5)	0	(0)	
Endometrioid	4	4	(3.4)	0	(0)		3	(2.5)	1	(0.8)	
LN metastasis						0.0009					0.7671
Yes	106	32	(1.1)	74	(46.2)		69	(58.0)	37	(31.2)	
No	13	10	(8.4)	3	(2.5)		9	(7.6)	4	(3.4)	
p53 status						0.0157					0.0221
Mutant	105	33	(27.7)	72	(61.0)		65	(54.6)	40	(33.6)	
Wild-type	14	9	(7.6)	5	(4.2)		13	(10.9)	1	(0.8)	
Clinicopathological factors	N	p21 expression, n (%)				P-value	ATF4 expression, n (%)				P-value
		Score≤6		Score>6			Score≤6		Score>6		
Total	119	116	(97.5)	3	(2.5)		39	(32.8)	80	(67.2)	
Age (years)						0.9833					0.0467
<50	39	38	(32.0)	1	(0.8)		8	(4.2)	31	(26.1)	
≥50	80	78	(65.5)	2	(1.7)		31	(26.1)	49	(41.2)	
FIGO stage						0.0003					0.0007
I–II	16	13	(10.9)	3	(2.5)		11	(9.2)	5	(4.2)	
III–IV	103	103	(86.6)	0	(0)		27	(22.7)	76	(63.9)	
Tumor histology						0.1981					0.2039
High grade serous	112	110	(92.4)	2	(1.7)		35	(29.4)	77	(64.7)	
Clear cells	3	3	(2.5)	0	(0)		0	(0)	3	(2.5)	
Endometrioid	4	3	(2.5)	1	(0.8)		4	(3.4)	0	(0)	
LN metastasis						0.0332					0.003
Yes	106	103	(86.6)	3	(2.5)		30	(25.2)	76	(63.9)	
No	13	11	(51.6)	2	(1.1)		9	(7.6)	4	(3.4)	
p53 status						0.0158					0.0075
Mutant	105	103	(86.6)	2	(1.7)		30	(25.2)	75	(63.0)	
Wild-type	14	12	(10.1)	2	(1.7)		9	(7.6)	5	(4.2)

## 4. Discussion

CCDC106 was first identified in a yeast two-hybrid assay but limited information is available about its cellular and molecular mechanisms [1]. Subsequent studies demonstrated that CCDC106 can promote proliferation and invasion in non-small cell lung cancer cells and in breast cancer cells [22,23]. Our findings support the notion that CCDC106 can regulate the progression of ovarian cancers. Moreover, we found that benign ovarian tumors express little to no CCDC106, but the expression of CCDC106 gradually increased in borderline and malignant ovarian tumors (Supplementary Figure 3). Thus, our data suggest that the overexpression of CCDC106 is associated with tumorigenesis and cancer progression.

Little is known about the mechanism that regulates CCDC106-induced tumor progression. Ning et al. demonstrated that in wild-type p53 Hela cells and MCF-7 breast cancer cells, p53 interacts with phosphorylated CCDC106 and promotes its degradation [33]. The degradation of p53 leads to the aberrant transcriptional activation of BCL-2, which leads to apoptosis escape of tumor cells [33]. In contrast, CCDC106 showed no biological alterations in MDA-MB-231 cells, a mutant p53 breast cancer cell line. Based on the above considerations, Ning et al. proposed that CCDC106 primarily promotes cancer growth by degrading wild-type p53 [33]. This is partially consistent with our findings. We previously reported that CCDC106 might increase the expression of cyclins via the phosphorylation of AKT both in A549 cells (wild-type p53) and in H1299 cells (mutant p53) [23]. Those data suggested that the CCDC106-induced tumor growth may not solely depend on the intrinsic apoptotic pathway, which is regulated by p53. In contrast, p53-independent regulatory mechanisms had not been characterized. Here we demonstrated that CCDC106 does not affect proliferation, invasion or EMT in A2780 (wild-type p53) cells (Figure 2e-h, Figure 3c, d, Figure 4c, d). In addition, we found that CCDC106 is predominantly localized in the cytoplasm of A2780 cells (Supplementary Figure 1). This suggested that the localization of CCDC106 and the presence of p53 determines the biological activity of CCDC106 and that cytoplasmic CCDC106 has no effect on the proliferation, invasion and EMT of wild-type p53 ovarian cancers.

To further assess this possibility, we evaluated the subcellular localization of CCDC106 in mutant p53 ovarian cancer cells (CAOV3 and SKOV3), in which CCDC106 is localized mainly in the nuclei, and in A2780 (wild-type p53) ovarian cancer cells that express CCDC106, which is primarily cytoplasmic. We found that CCDC106 induced the proliferation, colony formation and invasion of CAOV3 and SKOV3 cells while it had no effect on A2780 cells. The expression of cyclin- and EMT-related genes was in proportion to these findings. These data suggested that the function of CCDC106 is affected by its subcellular localization, namely, that the translocation of CCDC106 into nuclei is required to promote the growth and invasion of ovarian cancer cells, as suggested by Ning et al. [33].

Protein kinase CK2 is a conserved serine/threonine kinase. CCDC106 has two phosphorylation sites, S130 and S147, which bind to the CK2β subunit of CK2 kinase and are then phosphorylated by CK2 kinase. Ning et al. demonstrated that p53 interacts with phosphorylated CCDC106 and promotes its degradation in wild-type p53 Hela and MCF-7 cancer cells [33]. There is also a related study that a CK2 inhibitor regulates tumor phenotype by influencing the location of Zinc Finger CCCH-Type Containing (ZC3H8) protein at both promyelocytic leukemia (PML) bodies and Cajal bodies within the nucleus [37]. In view of the above studies, we found that the expression level of nuclear CCDC106 is significantly suppressed after treatment of SKOV3 cells with a CK2 inhibitor.

We identified ATF4 as a potential co-activator of CCDC106 in SKOV3 cells using mass spectrometry and data from four major databases (BioGrid, IntAct, MINT, STRING) and confirmed the expression level of ATF4 by western blot analysis (Figure 7a). A previous study verified that ATF4 is highly expressed in rhein derivative 4a treated ovarian cancer cells [38]. In line with this, our study demonstrated that ATF4 is significantly expressed in four ovarian cancer cell lines (Figure 7a). ATF4 is involved in promoting colony formation, progression, and metastasis in hepatocellular and lung carcinomas [39–41]. It has also been reported that ATF4 is an important regulator in p53-deficient cells in head and neck cancers [42]. Here, we also found that the knockdown of ATF4 suppressed colony formation and invasion in mutant p53 ovarian cancer cells (Figure 8c, d). Our immunohistochemistry results showed that ATF4 is strongly expressed in mutant p53 type ovarian cancer human samples (p = 0.00749) and a high ATF4 expression level is correlated with a poor prognosis in patients with ovarian cancers (p = 0.0045). In glioblastoma multiforme, studies demonstrated that ATF4 is a novel upstream regulator of p21 [36]. Consistent with that finding, our present study proved that silencing ATF4 considerably restrains the expression of p21, as well as downregulating the expression of p27 in SKOV3 cells (Figure 8a, b). We also demonstrated that ATF4 is a target of CCDC106 in SKOV3 cells (Figure 7c) and that the overexpression of CCDC106 downregulates ATF4 as well as p21 (Figure 8a, b). These results suggested that CCDC106 regulates p21 via the ATF4 pathway.

CCDC106 can modulate proliferation, colony formation and invasion only when it enters the nucleus, although it may regulate those activities through p53/BCL-2 (p53-dependent) and ATF4/p21 (p53-independent) mechanisms (graphical abstract).

## 5. Conclusion

The biological functions of CCDC106 depend not only on the functional state of p53, but also on the subcellular localization of CCDC106. Our study characterized a novel mechanism by which CCDC106 regulates the progression of mutant p53 ovarian cancers and suggests potential therapeutic targets for patients with p53-deficient ovarian cancers.

## Supplementary Material

Supplemental Material

## Data Availability

The data that support the findings of this study are openly available in figshare at https://doi.org/10.6084/m9.figshare.19524595,V1.
