# Supplementary figures and images for: CCDC106 promotes the proliferation and invasion of ovarian cancer cells by suppressing p21 transcription through a p53-independent pathway
Source: Bioengineered. 2022 Apr 29;13(4):10957–73. doi: 10.1080/21655979.2022.2066759 (PMC9208459; doi:10.1080/21655979.2022.2066759)

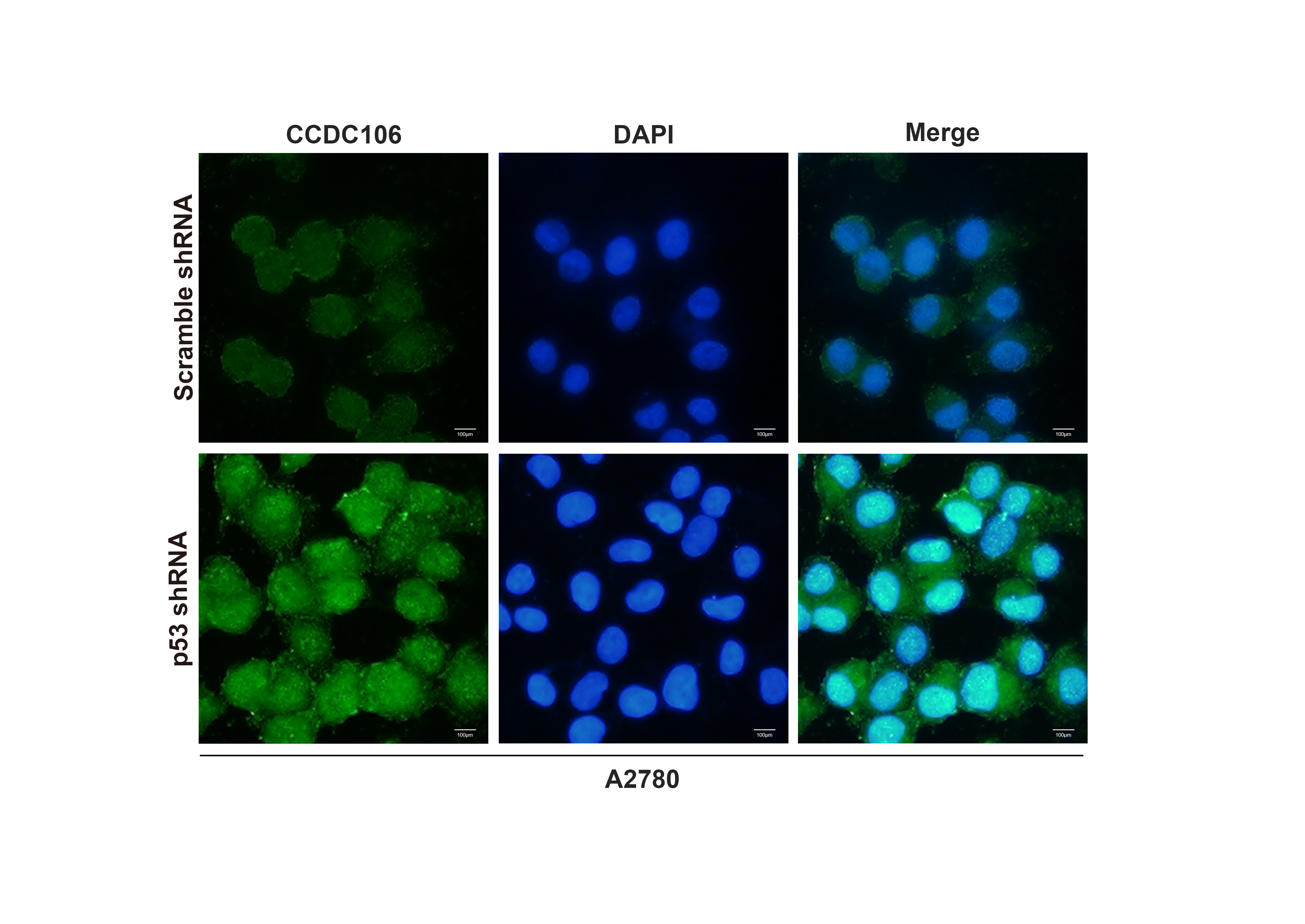

Supplement: Supplemental Material [file KBIE_A_2066759_SM7461.zip › supplementary/Supplementary figure 1.jpg]

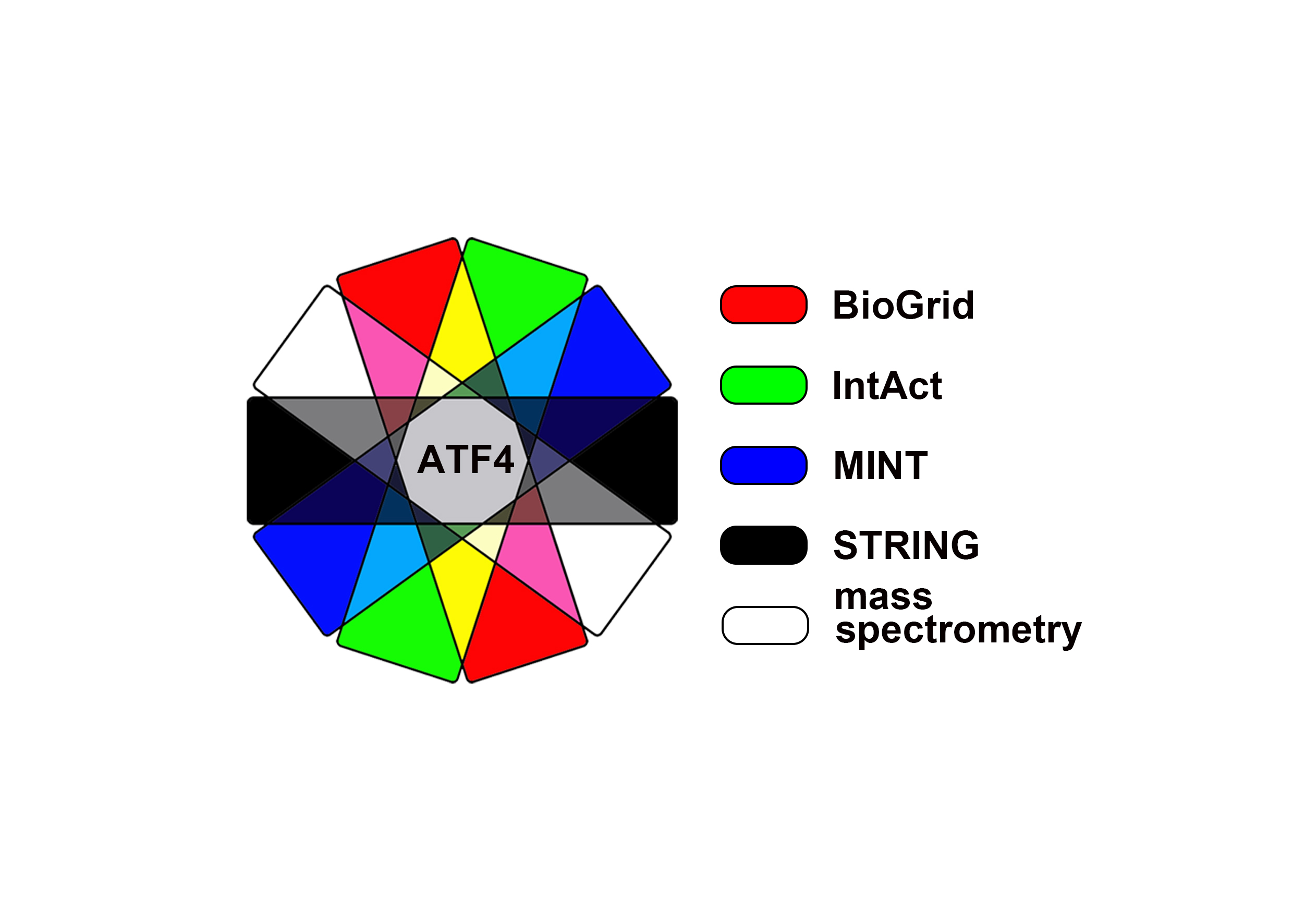

Supplement: Supplemental Material [file KBIE_A_2066759_SM7461.zip › supplementary/Supplementary figure 2.jpg]

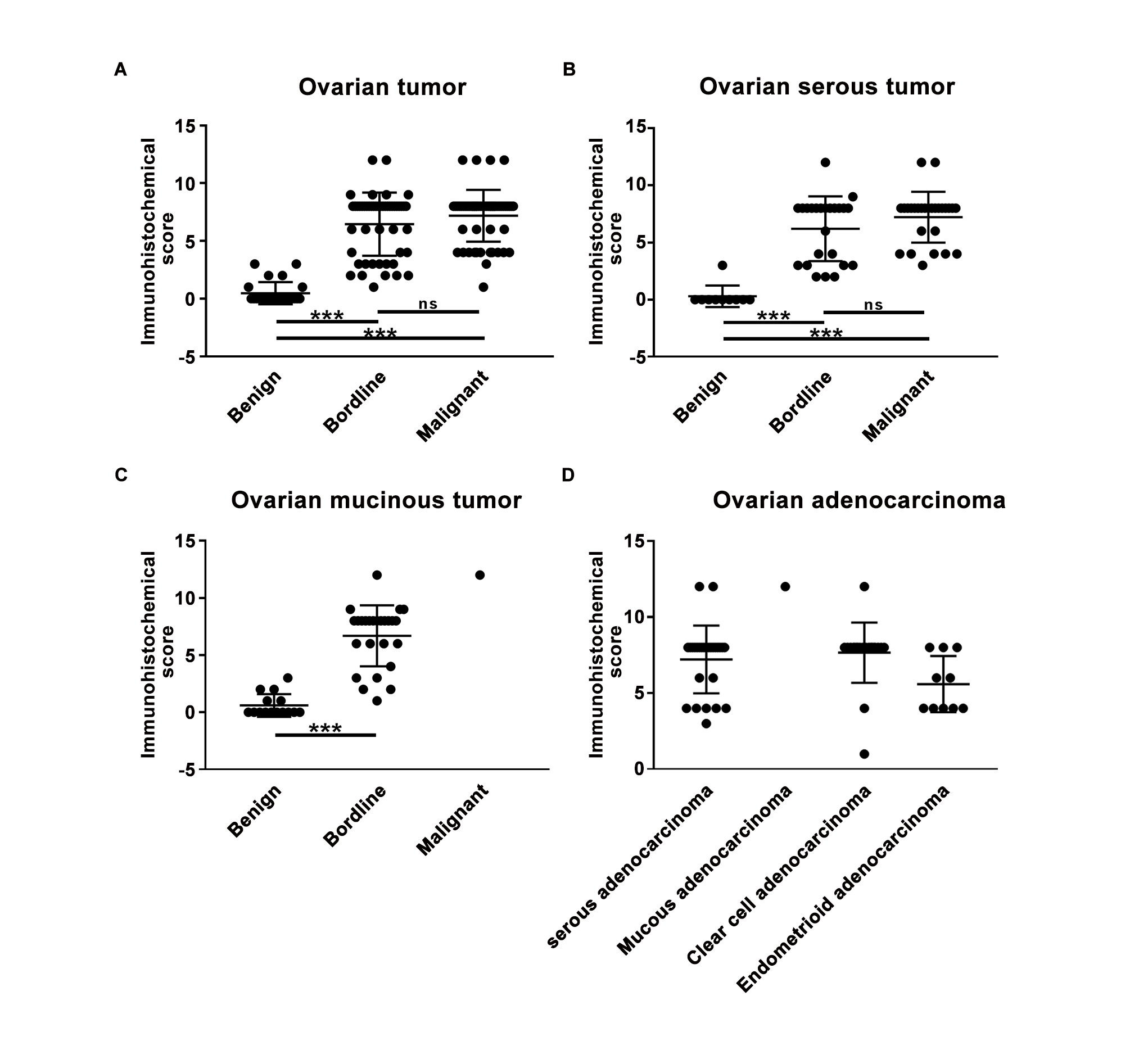

Supplement: Supplemental Material [file KBIE_A_2066759_SM7461.zip › supplementary/Supplementary figure 3.jpg]
